# Evidence-based decision-making support for determining the risk of schistosomiasis infection during large events in China: Application of risk assessment

**DOI:** 10.1371/journal.pntd.0013898

**Published:** 2026-01-09

**Authors:** Yuting Zuo, Gong Chen, Xin Mei, Xiao Xie, Yudan Song, Shuai Wang, Huatang Luo

**Affiliations:** 1 Wuhan Center for Disease Control and Prevention, Wuhan, China; 2 Chinese Center for Disease Control and Prevention, Beijing, China; 3 Medical Department, The Central Hospital of Wuhan, Tongji Medical College, Huazhong University of Science and Technology, Wuhan, China; 4 Zhuzhou Maternal and Child Health Hospital, Zhuzhou, China; University of Wisconsin-Madison, UNITED STATES OF AMERICA

## Abstract

**Background:**

To investigate the application of risk assessment in decision-making support for disease prevention and control during large-scale events involving schistosomiasis, a rapid system and process for assessing the risk of schistosomiasis transmission were developed.

**Method:**

A risk assessment indicator framework was developed through literature reviews and the Delphi method. Risk level determination was assessed using the Delphi method and a risk matrix method. Control measures were implemented based on the risk levels determination. Effectiveness evaluation was verified by monitoring schistosomiasis-related public health incidents and tracking key indicators—including oncomelania snail infection prevalence, snail density, human and livestock infection rates, wild feces detection, and sentinel mouse infection—over 1–3 years post-event, supplemented by repeated risk assessments.

**Result:**

The decision-making support system for schistosomiasis transmission prevention and control during large-scale events comprises a database of recent schistosomiasis cases, onsite schistosomiasis transmission risk monitoring, a case database of outbreaks, a database of assessment experts, a database of the distribution of oncomelania snail, a library of professional strategies, and a preplan. This system encompasses four processes, namely, identification of high-risk factors, risk assessment to determine areas with high, medium and low risk levels, implementation of targeted risk management and control measures, and evaluation of long-term effects.

**Conclusion:**

The risk assessment system was successfully applied to three large-scale events in Wuhan, effectively supporting evidence-based decision-making support and preventing schistosomiasis transmission. Post-event surveillance and reassessment of indicators verified the sustained effectiveness of the interventions. This closed-loop approach demonstrates that risk assessment is a vital tool for public health decision-making during large events in endemic areas.

## Author summary

Organizing large public events like festivals, construction projects, or international games in schistosomiasis-endemic regions requires proactive health risk management. In this study, we developed a decision-making support system for schistosomiasis transmission prevention and control during such events. Our framework integrates risk identification, risk assessment (using expert consultation, Delphi, and risk matrix methods), risk management and control, and effectiveness evaluation. We applied this system to three major events in Wuhan: the International River-Crossing Festival, the Wuhan Changjiang New Area development, and the Seventh World Military Games. We identified high-risk areas by analyzing epidemic surveillance data, oncomelania snail distribution, field risk monitoring, and population vulnerability (e.g., water sports venues near historical oncomelania snail environments). Targeted interventions—such as environmental hardening to eliminate snail habitats, increased sentinel mice monitoring, and public awareness campaigns—effectively prevented outbreaks. Our findings demonstrate that structured risk assessment provides actionable insights for health authorities. This approach balances safety with event feasibility, offering a scalable model for public health decision-making during large gatherings in endemic regions.

## Introduction

In recent years, China has witnessed an annual increase in large-scale public events, encompassing urban construction, water and soil development, and sporting events. These projects are characterized by short-term mass gatherings, high population mobility, complex risk factors, and intense social interactions [1]. These characteristics challenge the response capacity of local public health systems, necessitating proactive government identification and management of potential risks [2]. The 2008 Beijing Olympic Games marked a milestone by introducing risk management into public health security measures for major events [3], providing new paradigms for preventing and controlling public health risks [4]. Consequently, risk assessment—evaluating risk sources, the probability of occurrence, the degree of impact, categories, and management—has become an effective method for early warning, prevention, and control of public health emergencies [5].

Schistosomiasis, a waterborne infectious disease with complex transmission links, is significantly related to water systems and human activities [6]. Outbreaks have often been associated with water conservancy projects, land development, and recreational activities [7–9]. Evidence-based decision-making for schistosomiasis prevention and control during large-scale events differs substantially from routine prevention and control, requiring comprehensive consideration of goals and decision-making needs across different event stages [10]. Methods such as the analytic hierarchy process (AHP) and the risk matrix can provide clear, objective, and systematic results, making them particularly suitable for addressing vague and difficult-to-quantify problems, like schistosomiasis risk [11]. Therefore, control decisions for these events should be grounded in such risk assessments to ensure scientific rigor and effectiveness.

This study focuses on Wuhan, a city located in the middle and lower reaches of the Yangtze River, which was historically a highly endemic area for schistosomiasis. The cumulative number of patients reached 289,787, with a cumulative oncomelania snail area of 97,948.04 square metre [12]. Through decades of targeted interventions, including oncomelania snail control, comprehensive management, infection source control, and urbanization-driven environmental hardening improvements, the prevalence has decreased annually. Currently, 12 out of the 15 administrative districts remain endemic areas for schistosomiasis [13]. After 2017, the positive prevalence from fecal tests in the population was 0%, and the positive prevalence from blood tests was less than 0.1% [14]. By 2019, no locally acquired acute schistosomiasis cases had been reported for at least five consecutive years. However, recent large-scale projects, such as the construction of the Wuhan Changjiang New Area and the 7th World Military Games, have been located in these historically schistosomiasis-endemic areas, presenting renewed public health challenges.

Risk assessments is crucial for schistosomiasis prevention and control during large public events, particularly in endemic areas like the Yangtze River basin. However, while risk assessments for other communicable diseases have been conducted, the risk of schistosomiasis transmission in the context of short-term, large-scale gatherings remains understudied. Existing research and indicator systems are typically designed for long-term, general environmental assessments [15–17] and may not be suitable for short-term, specific, large-scale events. These systems often overlook critical factors such as population vulnerability and existing health safeguards during mass gatherings. Although some provinces have established preliminary systems, these primarily rely on traditional monitoring indicators and lack the sensitivity and specificity required for dynamic event risk assessment. Therefore, a targeted, rapid-response risk assessment system is needed to complement existing surveillance frameworks for large-scale events in endemic regions.

Initial development was investigated via descriptive qualitative analysis of outbreak data. For the period from 2008 to 2010, YE Chuchu et al. selected five provinces, including Hubei and Jiangsu, which encompass the Three Gorges Reservoir area and the South-to-North Water Transfer Project, respectively, to monitor potential schistosomiasis outbreak areas [17]. The risk of schistosomiasis transmission in these areas was analyzed in terms of transmission sources, importation of oncomelania snail, and dispersal potential [17]. The establishment of a domestic schistosomiasis transmission risk indicator system and the use of mathematical methods for systematic assessment have mostly occurred at the China Centre for Disease Control and Prevention (CDC) and in Jiangsu Province, respectively. For example, in 2017, staff at the CDC of Wuxi, Jiangsu Province, employed the literature search method to initially develop a framework for the risk assessment indicator system, scored and screened alternative indicators via the 2-round Delphi method, calculated normalized weights and combined the weights of indicators, established a risk assessment indicator system for Wuxi after schistosomiasis transmission interruption [18], and systematically assessed the risk level via the fuzzy comprehensive evaluation method, with a risk assessment score of 0.1936 (low risk). [18] On the basis of comprehensively analyzing the current status of schistosomiasis risk assessment research, the risk indicators for schistosomiasis transmission have become increasingly comprehensive, and only the weights of alternative indicators must be determined and assessed according to real-world local epidemic conditions. Moreover, an indicator system has been successfully established. However, with the gradual decrease in the number of outbreaks and advances in surveillance technology, the indicator system remains dominated by traditional surveillance indicators and is less likely to account for the impact of existing health security forces, with a decrease in sensitivity and quality. Infectious disease risk assessment methods such as the AHP, the fuzzy synthesis method, and the risk matrix method can provide clear and objective results, are systematic, can better address fuzzy problems and are suitable for various nondeterministic problems with complex influencing factors, but they are difficult to quantify. Currently, the risk of schistosomiasis transmission has mostly been analyzed subjectively and logically, with few objective quantitative assessments.

Wuhan, with its historical context of schistosomiasis and recent hosting of major events, provides an ideal environment for developing and testing targeted risk assessment systems. These events include: the annual International Cross-River Festival (involving open-water swimming), the large-scale development project of the Wuhan Yangtze River New Area, and the 2019 7th Military World Games (featuring aquatic events and attracting international participants). These activities offer diverse scenarios for assessing and managing schistosomiasis transmission risks. Every year, the 7.16 International River-Crossing Festival, a national festival, is held to commemorate Chairman MZD swimming across the Yangtze River. This festival includes timed races for competitive swimmers and untimed events for amateur enthusiasts. It attracts nearly 10,000 people from multiple countries and regions every year. The distance of the timed river-crossing race course is approximately 1.8 km from Mingkou Wharf in Wuchang District to Nan’an Zui in Hanyang District, and the distance of the untimed river-crossing course is approximately 6 km from Mingkou Wharf to Sanyang Square on the Jiang’an riverside. The International River-Crossing Festival is held in summer, and its relationship with disease occurrence is characterized primarily by the risk of waterborne disease transmission. As the event involves open-water swimming, participants may be at risk of contamination by pathogens such as bacteria, viruses, and parasites. Since 2017, the Wuhan government has planned the establishment of the Yangtze River New Town as a new national-level demonstration town. The size of the planned starting area is approximately 30–50 km2, and the size of the remote control area is 500 km2. The sites selected under the Yangtze River New Town programme are distributed mainly in Chenjiaji and Wuhu12. All seven villages within the starting area have been affected by schistosomiasis, and advanced and chronic schistosomiasis cases still occur. Furthermore, oncomelania snail environments exist along the Yangtze River, Sheshui River and Fuhe River. The 7th Military World Games were held in Wuhan in October 2019, and more than 10,000 people from over 100 countries and regions participated, making it the largest World Military Games to date15. In Wuhan, 35 venues were built, repaired and renovated. As of February 2019, approximately 100,000 urban construction craftsmen in Wuhan were involved in construction for the Military Games.

This study selected the Wuhan area in the middle and lower reaches of the Yangtze River as the research region, primarily based on its natural and historical factors closely associated with the prevalence of schistosomiasis. Wuhan features a dense river network and extensive beach marshlands, providing an ideal breeding environment for the sole intermediate host of Schistosoma japonicum—the Oncomelania snail—making it a historically high-endemic area for schistosomiasis within the Yangtze River Basin. Historically, the Yangtze River flood season inundated these beaches, facilitating the dispersal of snails via water flow. Concurrently, frequent water-contact activities among residents, such as farming, washing, and fishing along the riverbanks, significantly increased the risk of exposure to infested water, leading to the widespread prevalence of schistosomiasis in Wuhan. Although the current epidemic situation is effectively controlled, Wuhan remains a key surveillance and control region for schistosomiasis due to its typical hydrological and geographical characteristics of the Yangtze River Basin. Therefore, conducting research here holds significant practical importance and represents a valuable perspective.

Thus this study was conducted to establish and apply a rapid, general risk assessment methodology that supports decision-making for schistosomiasis prevention and control during mass public events. By systematically analyzing three case studies in Wuhan, we developed a targeted, rapid-response support system that complements existing surveillance frameworks. This system integrates dynamic risk identification, assessment, management, and outcome evaluation capabilities, providing a scalable model for theoretical foundation public health decision-making during large gatherings in schistosomiasis-endemic regions, filling a critical gap left by conventional long-term assessment approaches.

## Methods

### Ethics statement

This study was approved by the Ethics Committee of the Wuhan Center for Disease Control and Prevention (CDC), China (WHCDCIRB-K-2021052). All participants provided formal written informed consent. All the collected data were anonymous and self-administered.

### Framework of risk assessment

This study aimed to develop and apply a rapid, evidence-based risk assessment system to support decision-making for schistosomiasis prevention and control during large-scale public events in endemic areas. The framework consists of four interconnected stages, forming a closed-loop system: risk factor identification, risk level determination, risk management and control, and effectiveness evaluation. This structured approach integrates qualitative and quantitative methods to comprehensively assess transmission risks, prioritize interventions, and evaluate outcomes over time. The methodology was designed to be replicable in other schistosomiasis-endemic regions hosting similar events, as it builds on established national surveillance data, expert consensus techniques, and scalable risk matrix scoring.

### Risk factor identification

#### Literature review.

Literature searches and reviews were conducted in the PubMed database and the China Hospital Knowledge Database (CHKD), as well as in news media, for relevant documents and major public opinion trends concerning public health emergencies during large-scale events, such as the Olympics and World Expos, over the past 20 years. Based on the results of literature reviews, preliminary indicators of risk factor identification were extracted and generated in conjunction with the National Schistosomiasis Surveillance Programme issued by the Chinese Centre for Disease Control and Prevention.

#### Delphi method.

The Delphi method is a structured, iterative expert consultation technique that allows anonymous input and reduces individual bias. It is particularly suitable for complex, multi-factor health risk assessments where quantitative data are insufficient and expert judgment is essential. In China, health regulations require risk assessment for large-scale water-related events in schistosomiasis-endemic areas. The Delphi method is widely accepted, logistically feasible, and ethically straightforward to implement. We developed a questionnaire (S2 Appendix) and invited 11–15 experts with over 10 years of experience in schistosomiasis control from provincial, municipal, and academic institutions. The expert panel consisted of 13 members for the International River-Crossing Festival, 11 for the Wuhan Changjiang New Area, and 15 for the Seventh World Military Games. Through two rounds of anonymous scoring and feedback, consensus was reached on a set of risk indicators. Expert authority was assessed using the authority coefficient (Cr), where Cr ≥ 0.70 was considered acceptable. Coordination among experts was evaluated with Kendall’s coefficient of concordance (W), with P < 0.05 indicating significant agreement [19].

The final indicator system comprised two levels (Table 1). First-level indicators included: Routine Surveillance, Field Risk Monitoring, Population Vulnerability, and Preventability/Controllability. Second-level indicators were specific measures under each category, such as snail density, human infection rates, awareness levels, and resource availability.

**Table 1:** Risk assessment indicator database for large-scale activities.

First-level indicators	Second-level indicators
**Routine Surveillance Indicators**	Includes epidemic surveillance data and oncomelania snail data collected from the Schistosomiasis Monitoring System of the Chinese Center for Disease Control and Prevention: Infection prevalence for schistosomiasis in blood tests (local residents) Infection prevalence for schistosomiasis in blood tests (mobile populations) Infection prevalence for schistosomiasis in faecal tests (local residents) Infection prevalence for schistosomiasis in faecal tests (mobile populations) Infection prevalence for schistosomiasis in livestock Historical cumulative area of newly emerging oncomelania snail Existing oncomelania snail area Average density of live oncomelania snail Infection prevalence for schistosomiasis in oncomelania snail
**Field risk monitoring Indicators**	Whether an environment is suitable for oncomelania snail breeding Maximum density of live oncomelania snail Average density of live oncomelania snail Infection prevalence for schistosomiasis in oncomelania snails (crush microscopy) Infection prevalence for schistosomiasis in oncomelania snails (LAMP test) Infection prevalence for schistosomiasis in sentinel mice Infection prevalence for schistosomiasis in wild mice Infection prevalence for schistosomiasis of wild feces
**Population Vulnerability Assessment Indicators**	Real-time footfall Prevalence of awareness about schistosomiasis prevention and control Number of swimmers per time slot Inquiry detection rate
**Preventability and controllability indicators**	Includes control capacity data from the past five years on: Schistosomiasis funding over the past five years Quantity of material reserves Number of institutional personnel Prevalence of schistosomiasis emergencies Occurrence of schistosomiasis-related public opinion incidents
**Other short-term indicators**	Whether personnel originate from schistosomiasis-endemic areas Personnel health status Symptom monitoring

#### Routine surveillance indicators.

The past five years of routine surveillance data were collected from the Schistosomiasis Monitoring System of the Chinese Center for Disease Control and Prevention, including epidemic surveillance data and oncomelania snail data. Epidemic surveillance data include infection prevalence for schistosomiasis in blood/faecal tests among local residents and mobile populations and the infection prevalence for schistosomiasis in livestock in the surveillance villages. Three hundred local populations (aged 6 years and above) and two hundred mobile populations from surveillance villages, engaged in activities such as farming, aquaculture, or construction in snail-infested areas, were monitored. Serological screening was performed using the indirect haemagglutination test (IHA). Individuals who tested positive subsequently underwent etiological examination via the nylon bag egg hatching method (one stool, three tests) and the modified Kato-Katz thick smear method (one stool, three slides). For mobile populations, passive surveillance was also implemented through sentinel healthcare facilities, where suspected cases were screened serologically and referred for etiological confirmation when indicated. The positive prevalence for schistosomiasis in blood tests = number of individuals whose blood test for schistosomiasis was positive/number of individuals’ blood tests conducted; The positive prevalence for schistosomiasis in faecal tests = number of individuals whose faecal test for schistosomiasis was positive/number of individuals’ faecal tests conducted. As for the infection prevalence for schistosomiasis in livestock, during the autumn (September to November) each year, one hundred local grazing livestock (such as cattle and sheep) in surveillance villages were examined for schistosomiasis infection using the miracidium hatching method (one stool, three tests). The infection prevalence for schistosomiasis in livestock = the number of animals infected by schistosomiasis/the total number examined.

Oncomelania snail data include the historical cumulative area of newly emerging oncomelania snail, existing oncomelania snail area, the average density of live oncomelania snail, the infection prevalence for schistosomiasis in oncomelania snail [20]. Oncomelania snail data was conducted strictly in accordance with the Survey of oncomelanid snails (WS/T 563–2017) standard: field surveys adopted the systematic sampling combined with environmental sampling survey method. Parallel survey lines were set up in the survey environment (e.g., beach marshlands), with survey frames of 0.1 m^²^ placed at equal intervals of 10 m (frame distance). Additional frames were set up in specific environments where snails were likely to breed. A handheld Global Positioning System (GPS) was used to record the longitude and latitude of each survey environment. All snails within each frame were collected, placed into separate snail bags labeled with the environment name, type, frame number, and date. The crush method was used to identify snail viability (by observing contraction of soft tissues), and the crush microscopy method was employed to detect the presence of schistosomiasis miracidia or cercariae within the snails to determine infected oncomelania snail. Statistical indicators were calculated as: the infection prevalence for schistosomiasis in oncomelania snails was calculated as number of infected snails/ total number of dissected snails × 100%; the average density of living snails was calculated as total number of live snails collected/ total number of survey frames, with results expressed as “no./0.1 m²”.

#### Field risk monitoring indicators.

These indicators include whether an environment is suitable for oncomelania snail breeding, the average and maximum density of live oncomelania snail [21], the infection prevalence for schistosomiasis in oncomelania snails (including loop-mediated isothermal amplification (LAMP) test results) [22], the infection prevalence for schistosomiasis in sentinel mice, the infection prevalence for schistosomiasis in wild mice, and the infection prevalence for schistosomiasis of wild feces [23].

Whether an environment is suitable for oncomelania snail breeding is determined by the following criteria: oncomelania snails are amphibious creatures that thrive in moist, weed-infested areas with temperatures ranging from 20 to 25℃. In surveillance villages, five environments are selected for oncomelania snail monitoring, with at least 500 frames surveyed. A systematic sampling survey is applied to existing oncomelania snail habitats within the monitoring scope, with survey frames (0.1 m²) placed at equal intervals of 10 metre. If no oncomelania snails are detected, an environmental sampling survey is used to complete the investigation of that environment. For historical snail habitats and suspected breeding environments, an environmental sampling survey is conducted first; if snails are found, the survey switches to a systematic sampling survey. The longitude and latitude of each environment are recorded using a Global Positioning System (GPS). Collected snails are examined for viability, and the crush microscopy method is used to detect schistosoma infection. The loop-mediated isothermal amplification (LAMP) technique is applied to detect schistosoma nucleic acid in snail tissues (at least 500 snails per village, or all if fewer are available). Maximum/Average density.of living oncomelania snails = (Number of live snails collected/ number of survey frames) * 100%. Infection prevalence of schistosomiasis in oncomelania snails = (number of infected snails/ number of dissected snails) * 100%.

The survey of schistosoma infection in wild feces, where wild feces (including human, livestock, and other mammalian feces) were collected from oncomelania snail habitats, each sample placed in a labeled plastic bag and tested for schistosoma infection using the plastic cup hatching method (one stool, three tests) in accordance with GB/T 18640–2017 [24]; simultaneous survey of schistosoma infection in wild mice was conducted in historical, existing, or suspected oncomelania snail habitats using the snap‑trapping method over three consecutive nights with a minimum of 300 trap‑nights per environment, where captured mice were identified by species and examined for infection via mesenteric tissue microscopy, liver squash microscopy (one mouse, three tests), and liver homogenate microscopy, with any detection of schistosoma eggs, adult worms, or miracidia indicating infection; additionally, sentinel mice were deployed in high‑risk water bodies, retrieved after 30 days, and dissected to examine schistosoma infection; infection prevalence was calculated as follows: infection prevalence of schistosomiasis in wild feces = (number of positive wild feces samples)/ (total wild feces samples collected) * 100%; infection prevalence of schistosomiasis in wild mice = (number of infected wild mice)/ (total wild mice examined) * 100%; infection prevalence of schistosomiasis in sentinel mice = (number of infected sentinel mice)/ (total sentinel mice examined) * 100%.

#### Population vulnerability assessment indicators.

Population vulnerability assessment indicators are evaluated by real-time footfall, the prevalence of awareness about schistosomiasis prevention and control, and number of swimmers per time slot at epidemic surveillance village. A high-reliability questionnaire (S1 Appendix) was used to collect data about the population’s awareness of schistosomiasis prevention and control [25]. The questionnaire was developed based on the Schistosomiasis Prevention and Control Knowledge and Behavior Question Bank compiled by the National Institute of Parasitic Diseases, Chinese Center for Disease Control and Prevention. It included 10 single-choice questions on knowledge (e.g., awareness of schistosomiasis, its hazards, transmission routes, symptoms, treatment drugs, and susceptible seasons), 3 questions on attitudes (e.g., willingness to seek examination and treatment if infection is suspected, belief in the preventability and eradicability of schistosomiasis), and 3 questions on health practices (e.g., avoidance of contact with infested water, use of protective measures like rubber boots and ointment, and initiative to seek examination and treatment), totaling 16 items. The Cronbach’s α coefficient for the questionnaire was 0.766, indicating high reliability. A multi-stage stratified cluster random sampling method was employed on the project field. Data are collected through self-administered questionnaires under the supervision of two uniformly trained field investigators to ensure independent completion. The questionnaires were reviewed on-site and double-entered. Awareness prevalence/attitude holding prevalence/behavior formation prevalence (%) = Number of correct respondents/ Total number of respondents * 100%. The overall awareness holding/formation prevalence (%) = Σ number of correct responses per question/ (Total number of respondents * Number of questions) * 100%.

#### Preventability and controllability indicators.

The preventability and controllability indicators includes schistosomiasis funding over the past five years, quantity of material reserves, number of institutional personnel, prevalence of schistosomiasis emergencies, and occurrence of schistosomiasis-related public opinion incidents.

#### Other short-term indicators.

Other short-term indicators include whether personnel originate from schistosomiasis-endemic areas, their health status, and symptom monitoring.

### Risk level determination

An evaluation indicator system listed above was developed, and statistical charts were generated to serve as reference data for risk assessment.

#### Delphi scoring.

The same expert panel scored each identified risk factor based on two dimensions: probability of occurrence and severity of consequences. Scores ranged from 1 (lowest) to 5 (highest).

#### Risk matrix method.

The risk matrix is a visual, quantitative tool that combines likelihood and impact scores to prioritize risks. It is widely used in public health emergency management and is adaptable to event-specific scenarios. When combined with Delphi scoring, it balances expert judgment with structured analysis, enhancing objectivity and transparency. Building upon Risk Factor Identification, the risk analysis matrix framework outlined in the Risk Management Standard AS/NZS 4360:2004/ISO 31000 is employed in this method. The identified risk events were evaluated within a matrix based on two dimensions: the probability of occurrence and the severity of consequences. Each dimension was graded on a five-point scale. In this study, the influence of existing security and response capabilities were incorporated by adding an indicator for the controllable level (prevention and response capability) to the assessment framework. This approach aims to systematically review risk sources and influencing factors, reduce subjectivity in expert scoring, and achieve more comprehensive risk level determination. We used a 5*5 risk matrix (Table 2). The product of probability and severity scores yielded a risk score from 2 to 10, classified as low (2–4), medium (5–6), high (7–8), or very high (9–10) risk. Controllability was integrated as a modifier within the scoring framework. To derive risk scores, the experts scored the possibility, severity, and population vulnerability of risks [16] as Table 2.

**Table 2:** Results of the schistosomiasis transmission based on risk matrix method.

Possibility	Severity				
	Disastrous (5)	Serious (4)	Moderate (3)	Mild (2)	Negligible (1)
Unavoidable (5)	10	9	8	7	6
Highly possible (4)	9	8	7	6	5
Possible (3)	8	7	6	5	4
Improbable (2)	7	6	5	4	3
Rare (1)	6	5	4	3	2

Note: The risk level score ranges from 2–10, including a low risk (2–4), medium risk (5–6), high risk (7–8) and highest risk (9–10). The significance of these values is shown in Table 3.

#### Possibility (the probability of occurrence).

This factor was ranked as follows (from highest to lowest): unavoidable, highly possible, possible, improbable, and rare. Scoring considers dimensions such as epidemic status, transmission routes, population susceptibility, seasonal factors, and prevention/control capabilities.

#### Severity (the severity of consequences).

This factor was ranked as follows (from highest to lowest): disastrous, serious, moderate, mild, and negligible. The scoring system accounts for dimensions such as infectiousness, disease severity, and fatality prevalence.

A scoring database was created in Excel. The mean, standard deviation, and coefficient of variation (CV) of the scores were calculated. CV ≤ 20% indicates a low degree of dispersion within an individual score. The mean scores were analyzed to determine the risk level grade, with the significance of the levels detailed in Table 3.

**Table 3:** Risk level scoring criteria.

Risk level	Score	Score for different entities					
		Definition	Games	Government	Society	Global	Media
Very high	9–10	Enormous social impacts and losses; harm to human health	Cancellation or termination of the event	Notable influence on the international image; Considerable difficulty in risk communication; Beyond the capacity of health security; Direct government leadership is needed	People panic, affecting the normal social order	World Health Organization (WHO) travel warning	Close attention at home and abroad
High	7–8	Notable social impacts and losses; endangerment of human health	Directly affects the event	Notable influence on the international image;. Risk communication is difficult;. Health care requires multisectoral assistance	Affects the order of production and life	Travel advice	Domestic and foreign concerns
Medium	5–6	Certain social impacts and losses	Under control	Negligible impact on the international image; Effective risk communication; Health sector response	Affects local order of production and life	International community rumors	Minimal media attention
Low	2–4	Limited impacts on public opinion and population health	Business as usual	Business as usual	Business as usual	Business as usual	Business as usual

### Integration of methods

The Delphi method provided qualitative consensus and weighted indicators, while the risk matrix enabled quantitative prioritization. This combination allowed for a comprehensive, multi-perspective assessment that is both scientifically rigorous and operationally actionable.

### Risk management and control

On the basis of the assessed risk level (low, medium, or high), targeted graded risk management and control measures were implemented.

***High-risk areas***: Environmental modification, repeated snail control, increased monitoring frequency, public alerts, and restricted water access.
***Medium-risk areas***: Enhanced surveillance, sentinel monitoring, and health education.
***Low-risk areas***: Routine preventive measures.

All measures were documented and assigned to relevant local agencies for execution under the coordination of the Wuhan Center for Disease Control and Prevention.

### Effectiveness evaluation

#### Short-term and long-term evaluation strategy.

Given the time-limited nature of events, we designed a phased evaluation approach to assess both immediate and sustained impact:

***Short-term*** During and immediately after the event, we monitored for any schistosomiasis-related public health incidents.
***Long-term*** High-risk sites identified during the assessment were incorporated into special monitoring programs with increased frequency (e.g., monthly or bimonthly checks) in the year of the event. These sites were then integrated into routine annual surveillance for the following 1–3 years. Key indicators (e.g., snail density, infection rates) were tracked continuously.
***Re-assessment*** Periodic risk re-assessments were conducted to evaluate trends and intervention sustainability.

The risk assessments were organized and led by the Wuhan Center for Disease Control and Prevention, which convened expert panels, coordinated field investigations, and integrated multisource data.

## Results

### International river-crossing festival

The festival occurred during the flood season, which is a high-risk period for schistosomiasis. At the water entry point on Batan Road Beach in Wuchang District, oncomelania snail had been eradicated, and Wuchang District had achieved the standard for interrupting schistosomiasis transmission. However, both the upstream and downstream areas of the water body remain schistosomiasis-endemic zones with oncomelania snail habitats. The exit point of the timed race course on Hankou Riverside Beach exhibited no oncomelania snail detections in recent years. Upstream areas such as Dunan Island and Hannan Riverside Beach ranked among the highest in terms of the oncomelania snail density in Wuhan. In recent years, frequent floods have occurred. However, no positive oncomelania snails have been detected from the upstream areas extending to Wuchang or Hankou Riverside Beaches. The awareness prevalence of schistosomiasis prevention among recreational visitors exceeded 90%.

Via the use of the expert consultation method, the probability of positive oncomelania snail occurrence along the untimed river-crossing course during the festival was determined as low. The distances are short, the risks were deemed controllable, and the risk of schistosomiasis transmission was assessed as extremely low. Nevertheless, all areas along the river-crossing route contain oncomelania snail environments. Although no positive oncomelania snails were detected that year, recent epidemic data and field investigations suggested a potential infection risk for athletes and open-water staff, due to the long course. Located downtown with complex human traffic conditions, the riverside experienced significantly greater flow during the festival. A schistosomiasis transmission event could impact a large population via social activities, leading to a moderate risk level. Positive oncomelania snail occurrences have been found in the Phase II section over the past five years, and both human flow and oncomelania snail density were higher than those in the Phase I section. Consequently, the risk level was as follows: Phase II > Phase I.

Beyond large-scale events such as the International River-Crossing Festival, the persistence of oncomelania snail environments along the Hankou riverside impeded local schistosomiasis prevention efforts. Traditional drug-based oncomelania snail control could hardly achieve complete eradication without population rebound. Risk assessment indicated a moderate infection risk for the population. The government should gradually incorporate environmental upgrades for Phases II and I into urban construction plans and implement environmental hardening measures for oncomelania snail eradication. This should involve thorough pavement hardening of the beach surface and the creation of safe hydrophilic platforms. On the basis of spring oncomelania snail surveys, surveillance and oncomelania snail/ myracidium extermination were conducted again 1,000 m upstream of the starting point and 500 m downstream of the finish point to establish a safe water area. Inspection signs were posted in oncomelania snail environments, and patrols were deployed to deter unauthorized water contact. Prevention and control departments obtained participant information in advance, thereby enforcing strict adherence to the designated course. Postevent follow-up assessments (with consent) were conducted to identify potential schistosomiasis symptoms. No related schistosomiasis emergencies occurred during or after the festival. The government was subsequently encouraged to include improvements to marshland oncomelania snail habitats in the residential livelihood plan, investing 10 million yuan (equivalent to ~1.45 million USD) in rectification. The environment has since been fully hardened, with no oncomelania snail or schistosomiasis cases reported in recent years.

### Development of the Wuhan Changjiang new area

The research team predefined five schistosomiasis risk sections on the basis of water systems and the geographical locations of endemic villages: the Zhu Jiahe River section, the Chenjiaji section of the Hanbei River, the Wuhu section of the Hanbei River, the Chenjiaji section of the Yangtze River, and the Wuhu section of the Yangtze River. Data from 2012–2016 on epidemic surveillance, field risk monitoring, population vulnerability, and control levels were compiled into a risk assessment indicator database. The expert positive coefficient value was 100%. The expert authority coefficient (Cr) value exceeded 0.8. The value of Kendall’s coefficient of concordance (W) was 0.3 during the first round and 0.581 after the second round of assessment (P < 0.05). The average risk score for each section ranged from 1.8 to 2.6, indicating that all the sections could be classified as exhibiting low risk. The average scores were ranked as follows: Zhu Jiahe River section > Chenjiaji section of the Hanbei River > Chenjiaji section of the Yangtze River > Wuhu section of the Yangtze River > Wuhu section of the Hanbei River.

Risk management and control prioritized higher-risk sections. A segmented governance strategy was implemented based on the water system. The Zhu Jiahe River, an inland river less affected by surrounding areas, exhibits low governance costs and favorable effects. Recommendations included immediate dredging of river ditches and reconstruction/hardening of the water supply pipeline system to eliminate potential oncomelania snail breeding environments. Oncomelania snail breeding occurred in the Chenjiaji section. Adjacent areas of the Hanbei River exhibited high oncomelania snail density and frequent wild feces detections, with wide riversides attracting many recreational visitors. On the basis of the data, the recommendations included incorporating the Chenjiaji section of the Hanbei River into routine monthly risk monitoring plans; adding sentinel mouse, wild feces, and manure monitoring measures; and establishing additional patrol posts to discourage water contact. For the Yangtze River system, leveraging the construction of the Jiangbei Expressway to level the river bank by landfilling for oncomelania snail elimination was proposed. The Yangtze River and Hanbei River sections in the Wuhu area contained no oncomelania snail, with only chronic and advanced schistosomiasis cases reported. Enhanced population condition monitoring and case follow-up treatment are needed.

Effectiveness evaluation: The above policies were implemented, supplemented by regular dynamic risk monitoring and assessment. By 2022, when the entire Yangtze River New Town was completed and opened, risk assessments consistently indicated low risks. There were no risk events, such as oncomelania snail spread or new human/animal schistosomiasis infections. Suitable oncomelania snail breeding environments were completely remodeled, and the density of living oncomelania snail progressively decreased toward elimination.

### Seventh world military games

First round of assessment: 53 public health emergencies across four categories were evaluated: infectious diseases (including schistosomiasis), drinking water and public area hygiene, foodborne diseases, and vector biological hazards. Events with high occurrence probabilities included the occurrence of chikungunya fever, influenza, vector biological hazards, and norovirus. The average score for the possibility of local schistosomiasis outbreaks was 2.07 (ranking 36th), and the average severity of consequences was 2.93 (ranking 25th). A comprehensive analysis determined the probability of occurrence as improbable and the severity of consequences as moderate, resulting in an overall medium risk level.

In the second round of schistosomiasis-specific assessment, we combined data on venues, participant origins (including endemic countries), arrival/departure locations, event categories, and a city-wide oncomelania snail distribution map. Risk factors were defined as environments with current or historical oncomelania snail breeding sites within a 1-km radius of personnel activity or water-exposure trajectories. No current or historical oncomelania snail environments exist within 1 km of the Athletes’ Village or Media Center. Among 27 venues, one venue contained one current oncomelania snail environment, and six venues contained 13 historical oncomelania snail environments. Among the 107 accommodation hotels, two encompassed two historical environments, namely, ① Hannan District General Aviation Airport (Parachuting), ② Qingshan Riverside Beach Volleyball Center (Yangtze River), ③ Wuhan Air Force Airport, ④ National Fitness Center, ⑤ Dongxihu Sports Center (4 historical oncomelania snail sites), ⑥ Liangzi Lake (3 historical oncomelania snail sites), ⑦ Guobo Riverside (3 historical oncomelania snail sites), ⑧ Country Garden Hotel (accommodation for international dignitaries), and ⑨ Greenland Hotel (accommodation for competition staff). Considering the waterborne transmission of schistosomiasis, three water sports events in open or tidal environments connected to external water bodies were prioritized: sailing and open-water swimming in East Lake (Venue ①), beach volleyball (Venue ②), and the triathlon (Venue ⑥). Data on population vulnerability (captured) and control levels were incorporated into the risk assessment indicator database. Eleven experts evaluated these 10 key environments and events. The highest mean probability score for schistosomiasis transmission risk was obtained for Venue ① (score: 2.73; CV: 17.13%). The highest mean severity score of consequences was obtained for Hotel ⑧ (score: 3.73; CV: 12.53%). The risk levels were determined on the basis of the scoring results and subsequent consultation (Table 4, Table 5). The medium-risk category included Sites ①, ⑧, ②, ⑤, ⑥, and ⑦, while the low-risk category included Sites ③, ④, and ⑨.

**Table 4:** Results of expert scoring during the second round of the Delphi method.

Assessment Object	Possibility of Occurrence		Severity of Consequences	
	Mean Score	CV(%)	Mean Score	CV(%)
① Hannan District General Aviation Airport	2.73	17.13	2.82	14.35
② Qingshan Riverside Beach Volleyball Center	1.91	28.25	2.82	14.35
③ Wuhan Air Force Airport	1.27	36.7	2.55	32.22
④ National Fitness Center	1.36	37	3	0
⑤ Dongxihu Sports Center	1.82	22.25	3	0
⑥ Liangzi Lake	2.09	14.42	2.91	10.36
⑦ Guobo Riverside	1.73	37.44	3	0
⑧ Country Garden Hotel	1.36	37	3.73	12.53
⑨ Greenland Hotel	1.18	34.23	2.73	17.13
⑩ East Lake Sailing Venue	1.27	36.7	2.82	14.35
Kendall’s coefficient of concordance (W)	0.521 (*P* < 0.001)		0.507 (*P* < 0.001)	

NOTE: CV:Coefficient of Variation.

**Table 5:** Results of the schistosomiasis transmission risk matrix for the key environments and events.

Probability of occurrence	Severity of consequences				
	Disastrous (5)	Serious (4)	Moderate (3)	Mild (2)	Negligible (1)
Unavoidable (5)	10	9	8	7	6
Highly possible (4)	9	8	7	6	5
Possible (3)	8	7	6 (①)	5	4
Improbable (2)	7	6	5 (②, ⑤, ⑥, and ⑦)	4	3
Rare (1)	6	5 (⑧)	4 (③, ④, and ⑨)	3	2

Note: The risk level scores range from 2–10, including low risk (2–4), medium risk (5–6), high risk (7–8) and highest risk (9–10). The significance of these values is detailed in Table 3.

Risk management and control: The 10 key sites above were added to the annual risk monitoring tasks. The monitoring frequency was increased as follows: Venue ① underwent oncomelania snail (LAMP test) and wild feces monitoring at least monthly; the other venues were monitored at least bimonthly. Local governments comprehensively investigated suspicious environments in relevant venues or waters. Repeated oncomelania snail/ myracidium extermination activities were conducted in open-water competition areas. Specifically, one week before the competition, extermination targeted oncomelania snail and myracidium in the village hosting Venue ①. Schistosomiasis prevention and safety education involved installing appropriate warning signs and distributing multilingual leaflets with health tips, especially near oncomelania environments close to Hotels ⑧ and ⑨, with increased patrols to deter unauthorized water entry. Simultaneously, intensive schistosomiasis surveillance was implemented for patients with unexplained fever from Southeast Asia, Africa, and South America.

Effectiveness evaluation: Pre-event comprehensive inspections and monitoring revealed no risk events, such as the presence of infectious oncomelania snail or spread. No schistosomiasis-related risk events occurred during or after the games.

## Discussion

The risk assessment framework developed in this study complements, rather than replaces, the national schistosomiasis surveillance system by providing agility for time-bound events. As demonstrated in the three Wuhan case studies, this modular process leverages routine surveillance while incorporating real-time field data and event-specific vulnerabilities. The successful prevention of outbreaks underscores its value in shifting resources towards focused, preventive interventions.

In addition to the above cases, the researchers provided notable technical support for local schistosomiasis transmission prevention-related decision-making by assessing the risk during mass public projects/events in schistosomiasis endemic areas, such as river-to-lake diversion projects, the Dragon Boat Festival in Huangpi District, and the reconstruction of Tianxing Island. A risk assessment framework for schistosomiasis transmission prevention decision support was successfully established and included the following six modules. The schistosomiasis epidemic database reflects the long-term trends in population, livestock and oncomelania snail infections, with the village employed as the smallest unit, whereas field risk monitoring reflects short-term early warning information or real-time outbreak information. For early warning information or real-time epidemic conditions, the case database contained schistosomiasis-related emergencies and experiences, the expert database provided information on the areas of expertise and contact information of senior experts within this field, and the oncomelania snail distribution database provided the accurate locations of 2,109 environments in Wuhan city and visualized their information. The professional strategy library could facilitate the quick formulation of administrative decisions for the government, thus enabling risk assessment to guide technical support for rapid decision-making during large-scale activities impacted by schistosomiasis.

### Risk identification

The perfect risk management plan for mass public programs must include comprehensive information on the overall risk evaluation process and on each element of events [26]. Considering the complex epidemic history of schistosomiasis, which is influenced by natural, social, biological and other factors, the evaluator must not only clearly understand the natural history of schistosomiasis but also, more importantly, obtain data on the activity content, venue and trajectory of participants in advance; fully employ background information and known epidemic data to identify risk factors; and transform large, general surfaces into points that can be managed individually [27]. For example, the organization of the Yangtze River-Crossing Festival should focus on open-water races, timed race courses, and untimed river-crossing courses, which can be grouped as evaluation objects on the basis of their routes. The Yangtze River New Town government should focus on water and land utilization, which can be guided by the implementation of regional governance, and risk sections can be divided according to the geographical locations of rivers, lakes, swamps and villages in epidemic areas. Finally, the Military Games not only involve water and land use activities but also involve water sports, so risk source sections should be identified strictly according to the map of the distribution of oncomelania snail environments and the overlapping areas of venues or sporting events.

### Inclusion of evaluation indicators

At present, the index system for schistosomiasis is relatively mature. For example, in the South-to-North Water Diversion Project, the multidimensional comprehensive evaluation method and the expert consultation method were adopted to preliminarily determine surveillance and early warning indicators, which are largely influenced by transmission cases of oncomelania snail and supplemented by human and animal infections, for risk assessment of the East Route Project [28]. Moreover, a rapid assessment index system for high-risk schistosomiasis transmission environments in lake endemic areas was established via the Delphi method and the percentage weight method [29]. The governments of Jiangsu [30], Hubei [31], and Anhui [32] also established risk assessment index systems after schistosomiasis transmission occurrence, but the above fixed index system is suitable only for assessing the overall environment over a long period. For different types of short-term activities, such as large-scale urban construction, water conservancy development or sporting events, the importance of each index for different environments varies. Furthermore, there are certain shortcomings, such as low correlations with social factors and a lack of pertinence and applicability. Data should be shared with animal husbandry, forestry, quarantine, and sports departments according to the background of different competitions and the availability of suitable indicators to flexibly increase or decrease short-term early warning or syndromic surveillance indicators [17]; notably, a fixed indicator system should not be directly copied and adopted. Our research refers to the experience of predecessors, and brainstorming was conducted to summarize many large-scale activity experiences for establishing a two-level indicator library. In actual operations, to better meet the assessment needs for large-scale events, consultation and expert scoring should be employed to determine suitable indices for incorporation into the risk assessment system.

### Risk assessment methods and grading standards

Risk assessment results cannot rely solely on limited expert judgment, which may lack objectivity and comprehensiveness. To address this, our study employed structured methods to integrate diverse perspectives systematically. The Delphi method engaged 11–15 experts from provincial, municipal, and academic institutions across multiple disciplines (schistosomiasis control, military health, infectious disease, emergency management). Their expertise was validated through authority coefficients (Cr ≥ 0.70). Through multiple anonymous scoring rounds with feedback, consensus was reached, minimizing individual bias. Subsequently, the risk matrix method translated these qualitative judgments into quantitative scores (probability × severity), providing a visual and prioritized ranking of risks. This combined approach ensured a more objective, transparent, and comprehensive assessment than ad-hoc evaluation.

Expert consultation via conferences can be used for collective discussion among experts. According to the assessed objects and evidence, experts can fully use their knowledge and describe their experiences to obtain consistent risk assessment conclusions and management recommendations [33]. This method was adopted in risk management for public health emergencies at the 26th University in Shenzhen [27]. In recent years, expert meetings have gradually become routine at the early stages of determining indices to be included in risk identification.

In the risk matrix method, experts with certain authoritative and representative experience generally quantitatively score the identified risk factors in the two dimensions of the risk occurrence probability and hazard level and construct a matrix for the identified risk events to obtain risk scores [34]. At the 16th Asian Games in Guangzhou, empirical judgment, expert consultation, and the risk matrix method were employed to obtain the infectious disease risk level [35]. Researchers have considered the influence of the existing security force on risk and attributed it to the control ability level when obtaining a three-dimensional matrix [36]. In this study, the traditional two-dimensional matrix was still adopted, but the control ability was included in the indicator system data for experts to assess the possibility and severity of risk occurrence. In the logical framework, the risk matrix method is often employed in combination with other assessment methods. The authority and representativeness of the experts must also be evaluated. During the 8th Para Games (Hangzhou) in 2011, the Delphi method and risk matrix method were applied to determine the risk level of each infectious disease [37]. The Borda ordinal method aims to sort multiple risks at the same level. When the same risk level is relatively concentrated to form a risk knot, the risk matrix method and the Borda ordinal method can be adopted to comprehensively and successfully verify the vector risk, as was done at the 2016 World Horticultural Exposition [38]. The AHP aims to transform the overall judgment via the weights of multiple elements that constitute a complex problem into a pairwise comparison of these elements and then into a ranking judgment of the overall weights of these elements. An evaluation hierarchy model is established, and a risk judgment matrix is obtained. According to the relative importance and degree of influence of different risk behaviors relative to a certain criterion, quantitative assignments are performed, the value of the actual operation status of each indicator is normalized, and the weight of each layer, the total weight, the contribution degree of different risk factors and the overall risk occurrence level or risk priority level can be determined. In this study, the risk index system was established via the Delphi method and the AHP, and a risk matrix was constructed to obtain risk priority levels for different infectious diseases [15]. The study logic is rigorous and detailed, and the author explored the use of the AHP method to establish a risk assessment index system for monitoring sentinel rats after schistosomiasis elimination. At present, scholars have also applied more complex mathematical models, such as Bayesian, Markov chain prediction, and gray prediction models, for risk assessment [18], which has created new directions for research on schistosomiasis prevention and control in China.

In addition, since the measurement of the impact of risk sources on health, the economy, and society must be based on a literature search or survey data, the risk level classification of large-scale activities should be based on recognized standards or guidelines and a detailed summary of the literature.

## Strengths and limitations

The primary strength of this study lies in the development and real-world application of a closed-loop, evidence-based risk assessment system tailored for large-scale events in endemic areas. It successfully integrated routine surveillance, dynamic field monitoring, and expert consensus to guide targeted interventions, preventing schistosomiasis transmission across three diverse case studies. The framework is designed to be scalable and replicable. However, several limitations should be acknowledged. First, in public health practice, it is ethically and logistically unfeasible to establish untreated control groups during major events, making it challenging to definitively attribute the absence of outbreaks solely to the risk assessment. Second, the effectiveness of the assessment depends on data quality, expert availability, and local institutional capacity, which may vary. Third, while the risk matrix provides clear prioritization, the initial scoring still involves subjective expert judgment. Despite these limitations, this structured approach offers a superior, evidence-based alternative to unstructured decision-making, ensuring resources are focused on the highest risks.

## Conclusion

This study demonstrates that a structured risk assessment framework—combining a modular indicator library, Delphi expert consultation, and risk matrix analysis—effectively supports evidence-based decision-making for schistosomiasis prevention during large-scale events in endemic areas. Its application in Wuhan’s International River-Crossing Festival, Changjiang New Area development, and the 7th World Military Games successfully identified and mitigated transmission risks, with post-event surveillance confirming sustained control. We recommend that public health authorities in similar endemic regions adopt and adapt this closed-loop assessment model for planning large gatherings. Future efforts should focus on digitalizing the system for real-time data integration and expanding its application to other vector-borne diseases, thereby strengthening proactive health security for mass events.

## Supporting information

S1 Appendix High-reliability questionnaire of schistosomiasis transmission prevention. (DOCX)

S2 Appendix Workshop on risk assessment of schistosomiasis transmission in the start-up area of Yangtze River New Town, Wuhan City. (DOCX)
