# Supplementary material for: Evidence-based decision-making support for determining the risk of schistosomiasis infection during large events in China: Application of risk assessment
Source: PLoS Negl Trop Dis. 2026 Jan 9;20(1):e0013898. doi: 10.1371/journal.pntd.0013898 (PMC12788674; doi:10.1371/journal.pntd.0013898)
Supplement: S1 Appendix — (DOCX) [file pntd.0013898.s001.docx]

I. Basic Information

Age: __________

Gender: __________

Occupation: __________

Place of residence: __________

Any history of schistosomiasis: __________

II. Knowledge section

Have you ever heard of schistosomiasis?

A.Yes

B.No

Do you know the main ways through which schistosomiasis is transmitted?

A.Skin contact with contaminated water

B.Eating undercooked freshwater fish

C.Other (please specify) __________

3. Do you know that the area you live in or are visiting is an endemic area for schistosomiasis?

A.Yes

B.No

III. Beliefs and attitudes section

How much of a health risk do you think schistosomiasis is?

A.Very much

B.Quite large

C.General

D. Not too serious

Are you aware you live in or are now visiting a schistosomiasis-endemic area?

Yes

No

Not sure

IV. Behavioural component

Have you ever taken measures to prevent schistosomiasis?

A.Yes

B.No

Do you take protective measures (e.g., apply anthelmintic, wear gloves, wear rain boots, etc.) when you come into contact with water (e.g., swimming, playing in the water, mowing the lawn, fishing for fish, replenishing shrimps, etc.)?

A. I take protective measures every time I come into contact with water.

B. Occasionally take protective measures

C. Never take any protective measures
