# Supplementary material for: Evidence-based decision-making support for determining the risk of schistosomiasis infection during large events in China: Application of risk assessment
Source: PLoS Negl Trop Dis. 2026 Jan 9;20(1):e0013898. doi: 10.1371/journal.pntd.0013898 (PMC12788674; doi:10.1371/journal.pntd.0013898)
Supplement: S2 Appendix — (DOCX) [file pntd.0013898.s002.docx]

**Workshop on Risk Assessment of Schistosomiasis Transmission in the Start-up Area of Yangtze River New Town, Wuhan City**

Dear Experts,

Greetings! Thank you for attending the workshop on "Hygienic Evaluation of Schistosomiasis Transmission in the Construction of the New Yangtze River Town in Wuhan"!

On 17 July 2017, the Wuhan Municipal People's Government held a press conference to officially announce that the Yangtze River New Town will be located in the Chenjiayi-Wuhu zone in Hankou. The Wuhan Municipal Party Committee and Municipal Government have positioned the Yangtze River New Town as a city that adheres to world vision, international standards, Chinese characteristics, and high positioning, and builds the Yangtze River New Town as a model city that practices the new development concept. However, the two administrative districts of Jiangan and Huangpi, covered by the new town, are schistosomiasis-endemic areas. The construction of large-scale projects has brought significant challenges to preventing and controlling schistosomiasis while promoting economic development and social progress. The Wuhan Municipal Commission of Health and Family Planning (WMCHF) asked the Institute to carry out a hygiene evaluation of schistosomiasis in the Yangtze River New Town of Wuhan in July 2017, to make the new city more attractive to the public. In July 2017, the Wuhan Municipal Commission of Health and Planning asked our Institute to carry out a hygienic evaluation of the "New Town of Yangtze River" in Wuhan, aiming to make the construction activities of the new city in line with the national health policy and relevant regulations; to carry out a special assessment and demonstration work; to take risk management countermeasures in cooperation with the planning and construction units; and to synchronise the preventive control with the construction of the planning, to further promote the construction of the regional ecosystem and to ensure the public's health.

Given your rich experience and insights in this field, we invite you to be the consultant for the "Workshop on Hygienic Evaluation of Schistosomiasis in Wuhan Yangtze River New Town Construction".

The workshop will include the following three elements: ① Discuss the risk source indicators of the start-up area and evaluate the weight coefficients of their contribution to the risk of schistosomiasis transmission; ② Evaluate the risk of acute schistosomiasis infections in the start-up area through the results of various risk source data obtained from the past years and on-site investigations in each water system area; ③ Propose the risk sources of schistosomiasis transmission in the process of the construction of future new cities and evaluate their risk weights by interpreting the plan and searching the literature. Risk sources in the future construction of the new town are proposed through the interpretation of the plan and the search of literature, and their risk weights are assessed.④ Based on the defined risk level, the prevention and control countermeasures will be discussed.

Thank you again for your strong support and hard work!

Basic information table of experts

No. of experts________

Type of expert: A. Health administration B. Blood defence expert (please type√ )

| Name |  | Sex |  | Age |  | Highest academic qualification |  |
| --- | --- | --- | --- | --- | --- | --- | --- |
| Work unit |  | | | | | Title |  |
| Address |  | | | | | Postcode |  |
| Phone number |  | | | Email |  | | |
| Engaged in the profession: | | | | | Years in the profession: | | |
| Main or Interested Research Direction: | | | | | | | |

Risk factors for schistosomiasis transmission in the starting area of Yangtze River New Town.

| First-level weighting indicators | | | | | | Weighting coefficient of second-level indicators | | | | | |
| --- | --- | --- | --- | --- | --- | --- | --- | --- | --- | --- | --- |
|  | Zhujiahe | Chenjiayi Area of the Hanbei River | Hanbei River Wuhu Area | Chenjiaji Area of the Yangtze River | Yangtze River Wuhu Area |  | Zhujiahe Area | Chenjiaji Area of the Hanbei River | Hanbei River Wuhu Area | Chenjiaji Area, Yangtze River | Yangtze River Wuhu Area |
| Oncomelania Situation |  |  |  |  |  | A1.The cumulative area of newly discovered oncomelanias in history |  |  |  |  |  |
|  |  |  |  |  |  | A2. Area with oncomelanias detected in the same year |  |  |  |  |  |
|  |  |  |  |  |  | A3. Average density of live oncomelanias |  |  |  |  |  |
|  |  |  |  |  |  | A4. oncomelania infection rate (microscopic examination) |  |  |  |  |  |
|  |  |  |  |  |  | A5. oncomelania infection rate (LAMP method) |  |  |  |  |  |
| **10 10 10 10 10** | | | | | | | | | | | |
| Population infection Situation |  |  |  |  |  | B1. Population infection rate |  |  |  |  |  |
|  |  |  |  |  |  | B2. Total number of patients in the year |  |  |  |  |  |
|  |  |  |  |  |  | B3. Historical cumulative number of new patients detected |  |  |  |  |  |
| **10 10 10 10 10** | | | | | | | | | | | |
| Wild rats |  |  |  |  |  | C1. Number of wild rats |  |  |  |  |  |
|  |  |  |  |  |  | C2. Prevalence of infection in wild rats |  |  |  |  |  |
| **10 10 10 10 10** | | | | | | | | | | | |
| Dung |  |  |  |  |  | D3. Number of wild feces |  |  |  |  |  |
|  |  |  |  |  |  | D4.Positive wild faeces |  |  |  |  |  |
| **10 10 10 10 10** | | | | | | | | | | | |
| Sentinel rodents |  |  |  |  |  | E5. Sentinel rat infection rate |  |  |  |  |  |
| **10 10 10 10 10** | | | | | | | | | | | |
| Prevention and control strength |  |  |  |  |  | F1.Control level |  |  |  |  |  |
|  |  |  |  |  |  | F2.Emergency response capacity |  |  |  |  |  |
|  |  |  |  |  |  | F3.Public Awareness of Haematological Control |  |  |  |  |  |
|  | **10** | **10** | **10** | **10** | **10** |  | | | | | |

Note: The sum of the weights of the indicators is equal to 10, the weight coefficients of the first-level indicators add up to 100, and the sum of the weight coefficients of the second-level indicators corresponding to each first-level indicator adds up to 100.

### 1.Risk matrix method

Based on the risk analysis matrix method of the Risk Management Standard AS/NZS4360:2004/ISO 31000 (Australia-New Zealand Standard), a matrix is constructed by experts on the identified risk events from the dimensions of probability of occurrence of risk and level of harm; a risk score is obtained by scoring the likelihood of the risk, the damage and the vulnerability of the population. This assessment takes into account the impact of existing safeguard forces on risk, adds indicators for the controllable level of risk occurrence, and systematically sorts out the sources of risk and related influencing factors, to reduce the subjective impact of the experts' scores, and to judge the level of risk more comprehensively. Three factors are positively correlated with the level of risk: the likelihood of the risk occurring, the hazard, and the public health vulnerability of the population, and one factor is negatively correlated: the level of controllability (preventive treatment capacity).

**Risk matrix classification table**

| Accident (incident) Likelihood of occurrence | Degree of impact of the occurrence of the accident (incident) | | | | |
| --- | --- | --- | --- | --- | --- |
|  | Very serious (5) | Severe (4) | Moderate (3) | Minor (2) | Negligible (1) |
| Necessary (5) | 10 | 9 | 8 | 7 | 6 |
| Very likely (4) | 9 | 8 | 7 | 6 | 5 |
| Possible (3) | 8 | 7 | 6 | 5 | 4 |
| Unlikely (2) | 7 | 6 | 5 | 4 | 3 |
| Rare (1) | 6 | 5 | 4 | 3 | 2 |

Note: Risk score 2-10, where L - low hazard risk (2-4), M - medium hazard risk (5-6), H - high hazard risk (7-8), E - very high hazard risk (9-10).

1. Hazardousness: The hazardousness of the occurrence of acute time in the start-up area of Changjiang New Town under the current status of the epidemic is assessed by analysing the geographic scope of the risk impact, the number of people affected, the economic loss caused, the severity of the effect on the health of the population, the degree of damage to the critical infrastructures or ecological systems, the impact on the stability of the society and the credibility of the government, as well as the psychological pressure on the public.

**Hazard Assignment and Meaning**

| **Hazard level** | **Value** | **Meaning** |
| --- | --- | --- |
| Very serious (1) | 9-10 | Once the risk occurs, it will cause significant social impact, endanger the population's health, and cause substantial losses. |
| Serious (2) | 7-8 | Once the risk occurs, it will cause significant social impact, endanger the health of human beings, and cause substantial damage. |
| Moderate (3) | 5-6 | Once the risk occurs, it will cause some social impacts and some losses |
| Slight (4) | 3-4 | Once the risk occurs, it will cause minor health impacts on social opinion and the population |
| Negligible (5) | 0-2 | Once the risk occurs, there is no impact on the main work carried out or the population |

② Likelihood: Based on the various risk factor indicators identified, combined with the assessment background of this study and the results of epidemic surveillance in previous years, assess the likelihood of an acute sensory event occurring in the start-up area of Cheung Kong New Town under the current epidemic status.

**Likelihood Assignment and Meaning**

| **Likelihood level** | **Range** | **Meaning** |
| --- | --- | --- |
| Inevitable(5) | 9-10 | Very high frequency of occurrence within the assessment |
| Very likely to occur(4) | 7-8 | Higher frequency of occurrence within the assessment |
| Likely to occur(3) | 5-6 | Has not occurred in the assessment area, but occurs with high frequency in similar areas |
| Unlikely to occur(2) | 3-4 | Has not occurred within the assessment area but occasionally in similar areas. |
| Rare(1) | 0-2 | Has not occurred within the scope of assessment, and rarely occurs in similar regions |

③Public health vulnerability of the population: judged according to the degree of tolerance of the population facing the hazard, which can be considered in terms of the susceptibility of the population, the public's psychological tolerance, the public's public health awareness, and the ability of self-rescue and mutual aid. It is divided into five levels: very low, low, medium, high, and very high.

**Vulnerability Assignment and Meaning**

| **Vulnerability Level** | **Range** | **Meaning** |
| --- | --- | --- |
| Very high (5) | 9-10 | Very likely to cause morbidity or mortality in the population |
| High (4) | 7-9 | More likely to cause morbidity or mortality in the population |
| Moderate (3) | 3-7 | Easily causes morbidity or mortality in the population |
| Low (2) | 1-3 | Rarely causes morbidity or mortality in the population |
| Very low (1) | 0-1 | Will not cause morbidity or mortality in the population |

④ Controllable level: The controllable level (preventive and disposal capacity) of acute sensation in the start-up area of Changjiang New Town under the current status of the epidemic can be evaluated from the considerations of the completeness of institutional monitoring system, emergency prevention and control force, medical rescue capacity, technical reserves, health resources, public health infrastructure, and so on.

**Controllable level assignment and meaning**

| **Controllability level** | **Assignment range** | **Meaning** |
| --- | --- | --- |
| 5 | 9-10 | Easier to control |
| 4 | 7-8 | Controllable |
| 3 | 5-6 | Harder to control |
| 2 | 3-4 | Difficult to control |
| 1 | 0-2 | Uncontrollable |

**Instructions for filling in the form:**

For ① ② ③④ scoring, the scoring assignment standard is as in the above table.

**Risk Assessment of Schistosomiasis Transmission in the Start-up Area of Changjiang New Town**

|  | **Risk area** | **①Hazard**  **（I）** | **②Likelihood**  **（L）** | **Hazard probability of occurrence（H=I*L）** | **③Vulnerability（V）** | **④Controllability**  **（AC）** | **Risk score**  **R =H×V-AC** | **Risk Matrix Rating** |
| --- | --- | --- | --- | --- | --- | --- | --- | --- |
| Overall Assessment | Zhujiahe Area |  |  |  |  |  |  |  |
|  | Chenjiayi Area of the Hanbei River |  |  |  |  |  |  |  |
|  | Hanbei River Wuhu Area |  |  |  |  |  |  |  |
|  | Chenjiaji Area of the Yangtze River |  |  |  |  |  |  |  |
|  | Wuhu Area of the Yangtze River |  |  |  |  |  |  |  |
| Spreading and infection of oncomelanias | Zhujiahe Area |  |  |  |  |  |  |  |
|  | Chenjiayi Area of the Hanbei River |  |  |  |  |  |  |  |
|  | Hanbei River Wuhu Area |  |  |  |  |  |  |  |
|  | Chenjiaji Area of the Yangtze River |  |  |  |  |  |  |  |
|  | Wuhu Area of the Yangtze River |  |  |  |  |  |  |  |
| Increase in the infection rate of the population and the emergence of acute sensations | Zhujiahe Area |  |  |  |  |  |  |  |
|  | Chenjiayi Area of the Hanbei River |  |  |  |  |  |  |  |
|  | Hanbei River Wuhu Area |  |  |  |  |  |  |  |
|  | Chenjiaji Area of the Yangtze River |  |  |  |  |  |  |  |
|  | Wuhu Area of the Yangtze River |  |  |  |  |  |  |  |
| Positive for wild rats, wild faeces, and sentinel rats | Zhujiahe Area |  |  |  |  |  |  |  |
|  | Chenjiayi Area of the Hanbei River |  |  |  |  |  |  |  |
|  | Hanbei River Wuhu Area |  |  |  |  |  |  |  |
|  | Chenjiaji Area of the Yangtze River |  |  |  |  |  |  |  |
|  | Wuhu Area of the Yangtze River |  |  |  |  |  |  |  |
| Overloaded prevention and emergency response capacity, and weak public awareness of blood prevention | Zhujiahe Area |  |  |  |  |  |  |  |
|  | Chenjiayi Area of the Hanbei River**区** |  |  |  |  |  |  |  |
|  | Hanbei River Wuhu Area |  |  |  |  |  |  |  |
|  | Chenjiaji Area of the Yangtze River |  |  |  |  |  |  |  |
|  | Wuhu Area of the Yangtze River |  |  |  |  |  |  |  |
